# Tumors of the Central Nervous System: An 18-Year Retrospective Review in a Tertiary Pediatric Referral Center

**Published:** 2015

**Authors:** Hosein AGHAYAN GOLKASHANI, Hossein HATAMI, Abdonaser FARZAN, Hassan Reza MOHAMMADI, Yalda NILIPOUR, Maliheh KHODDAMI, Farzaneh JADALI

**Affiliations:** 1 Department of Neurosurgery, Loghman Hospital, Shahid Beheshti University of Medical Sciences, Tehran, Iran; 2 Department of Public Health, School of Health, Shahid Beheshti University of Medical Sciences, Tehran, Iran; 3 Department of Neurosurgery, Mofid Hospital, Shahid Beheshti University of Medical Sciences, Tehran, Iran; 4 Pediatric Pathology Research Center, Mofid Children’s Hospital, Shahid Beheshti University of Medical Sciences, Tehran, Iran

**Keywords:** Pediatric brain tumors, Pediatric spinal tumors, Demography, Trends

## Abstract

**Objective:**

Few studies exist on the demographics and trends of pediatric central nervous system (CNS) tumors in Iran. In this study, we retrospectively reviewed all cases with confirmed CNS tumors admitted to Mofid Pediatric Hospital, Tehran, Iran during the last 18 years.

**Materials & Methods:**

Data on gender, age of diagnosis, pathologic classification and tumor location were extracted from the available medical records. We used the last version of International Classification of Childhood Cancer.

**Result:**

Overall, 258 (81.9%) brain tumors and 57 (18.1%) spinal tumors were identified. Our subjects comprised of 147 (46.7%) female and 168 (53.3%) male children. More male dominancy was observed in brain tumors with a male to female ratio of 1.2 compared with 1.03 of spinal tumors. Malignant CNS tumors were most common in 1-4 yr age group. The four most common brain tumors in our subjects were astrocytomas, medulloblastoma, ependymoma and craniopharyngioma. Overall, 53.1% of the brain tumors were supratentorial. Gliomas, PNET and neuroblastma were the most frequent primary spinal tumors in our study. We observed an increasing trend for both brain and spinal tumors that was moreremarkable in the last 5 years.

**Conclusion:**

Our results are comparable with similar single center studies on CNS tumors during childhood. The observed disparities could be attributed to the single center nature of our study and geographical, environmental and racial variations in pediatric CNS tumors. The increasing trend of both brain and spinal tumors could warrant further investigations at provincial and national levels to investigate probable contributing environmental risk factors.

## Introduction

Brain tumors are the most common solid malignancy during childhood. Moreover, neoplasms of central nervous system (CNS) are the second leading malignancy in pediatric population, following tumors of the hematopoietic and lymphoid tissues (1, 2). These malignancies pose a significant burden to the health system and finding more cost-effective diagnostic and therapeutic management options is of utmost importance. Use of novel diagnostic and therapeutic options based on advanced molecular approaches has been promising in this regard (3). However, despite notable technological improvements, optimal management of childhood CNS malignancies remains a significant challenge in pediatric neuro-oncology, particularly in the less developed countries (4, 5). Various epidemiologic investigations on pediatric CNS malignancies have been published, highlighting the possible geographical and racial variations of these diverse disease categories (6-8). Use of different classification methodologies in cancer registries and change of tumor subtypes over time have further complicated the interpretation of observed variations among different investigations. In addition, some studies have reported increasing trends of childhood brain tumors; yet this increased incidence has been attributed to the improved diagnostic capabilities rather than an actual change in annual incidence rate (9). Few studies exist on the demographics and trends of pediatric CNS neoplasms in Iran. In this study, we retrospectively reviewed all cases of CNS tumors admitted to Mofid Pediatric Hospital during the last 18 years. Our results on demographic variations of different tumor categories and trends of CNS tumors could provide further insight to implement more evidence-based policies regarding pediatric neurooncology in Iran.

## Materials & Methods

In this study, we extensively reviewed all cases with confirmed diagnosis of CNS tumors admitted to Mofid Pediatric Hospital, Tehran, Iran from March 1996 to March 2013. Data on gender, age of diagnosis, pathologic classification and location of tumors were extracted from the available medical records. We used the last published version of International Classification of Childhood Cancer (ICCC), used by The Surveillance, Epidemiology, and End Results (SEER) program in the United States (10). This classification system is based on tumor morphology and primary site, with an emphasis on morphology. In cases of altered diagnostic categories, pathologic blocks were re-examined by a specialist in pediatric pathology and tumors were reclassified accordingly. Moreover, in cases of diagnostic uncertainty, diagnosis was confirmed by immunohistochemistry (IHC). All duplicate cases and those with incomplete data or uncertain pathologic diagnosis were excluded. Statistical analyses were performed using SPSS version 16 (Chicago, IL, USA). For categorical and continuous variables, Chi-square and student t-test were used respectively. P-values less than 0.05 were regarded as statistically significant. Institution Review Board (IRB) in Pediatric Infectious Research Center approved the study protocol according to the Declaration of Helsinki.

## Results

**Fig 1:**
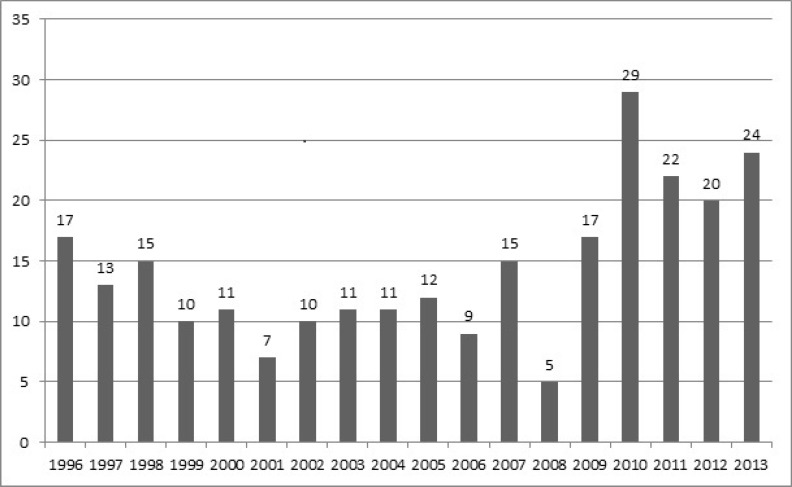
Trend of pediatric brain tumors

**Fig 2:**
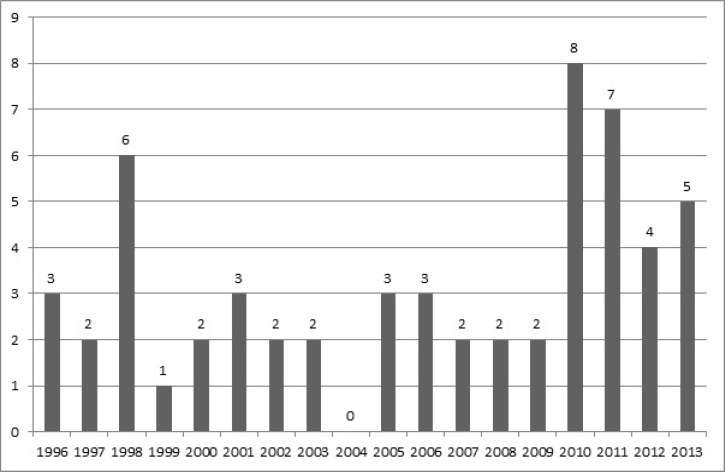
Trend of pediatric spinal tumors

In this study, we managed to collect demographic data on 315 pediatric CNS tumors during the last 18 years. Overall, 258 (81.9%) brain tumors and 57 (18.1%) spinal tumors were identified. Our subjects comprised of 147 (46.7%) female and 168 (53.3%) male children with an overall 1.14 male to female ratio. Gender distribution was 117 (45.3%) females vs. 141 (54.6%) males and 28 (49.1%) females vs. 29 (50.1%) males for brain and spinal tumors, respectively. More male dominancy was observed in brain tumors with a male to female ratio of 1.2 compared with 1.03 of spinal tumors. The mean (SD) age of diagnosis was 5.5 yr (4.0) and 5.2 yr (4.3) for subjects with brain and spinal tumors, respectively. Both brain and spinal tumors were more frequent in 1-4 year age group with the following frequencies, 89 (34.5%) and 23 (40.4%) in that order. Mean age of diagnosis did not significantly differ between genders. Gliomas were the most common diagnosed primary brain tumor among the subjects and frequency of different subtypes was as follows: ependymoma 32 (12.4%), pilocytic astrocytoma 28 (10.9%), low grade astrocytoma 26 (10.1%), glioblastoma multiforme 9 (3.5%), unspecified gliomas 8 (3.1%), mixed neuronalglial tumors 6 (2.3%), ependymoblastoma 5 (1.9%), astroblastoma 1 (0.4%) and oligodendroglioma 1 (0.4%). Following gliomas, embryonal tumors were the second leading primary brain tumor among the subjects with the following frequencies: medulloblastoma 52 (20.2%), Primitive neuroectodermal tumour (PNET) 16 (6.2%) and rhabdoid tumors 3 (1.2%). Other primary brain tumors were observed with following order of frequency: craniopharyngioma 17 (6.6%), meningioma 12 (4.5%), choroid plexus papilloma 8 (3.1%), lymphoma 7 (2.7%) and pineal parenchymal tumors 5 (1.9%). Overall, 19 (7.4%) metastatic brain tumors were identified and the most common source of metastasis were neuroblastoma and malignant germ cell tumors. Most common brain tumors were most frequent in 0-3 year age group, 19 (30.1%) of astrocytoma, 21 (40.4%) of medulloblastoma and 18 (56.2%) of ependymoma cases were detected in 0-3 yr age groups. The average age of diagnosis and gender distribution of different brain tumor categories are listed in Tables 1 and 2. Overall, 137(53.1%) and 121 (46.9%) of brain tumors were supratentorial and infratentorial, respectively. Most common supratentorial brain tumors were astrocytoma 22 (18.2%), craniopharyngioma 17 (14%) and PNET 16 (13.2%). Most common infratentorial tumors were medulloblastoma (38%), astrocytoma 41 (30%) and ependymoma 28 (20.5%). Gliomas were also the most common diagnosed primary spinal tumor among the subjects with the following observed frequency in different pathologic subgroups; astrocytoma 5 (8.8%), unspecified gliomas 3 (5.3%), pilocytic astrocytoma 2 (3.5%), ependymoma and ependymoblastoma with similar frequency of 1 (1.8%). Following gliomas, PNET and neuroblastoma were the most common primary spinal tumors with similar frequency of 7 (12.2%), followed by meningioma with frequency of 6 (10.5%). Metastatic spinal cancers were common and 19 (33.3%) of these cases were identified mostly metastasized from neuroblastoma, germ cell tumors, Ewing’s sarcoma and rhabdomyosarcoma. Regarding the age of diagnosis of most frequent spinal tumors, 3 (42.9%) of astrocytic tumors were distributed in 0-3 and 9-12 yr age groups, 3 (42.9%) of PNET cases and 4 (66.7%) of meningiomas were detected in 6-9 and 12-15 yr age groups, respectively. The average age of diagnosis and gender distribution of subjects with spinal tumors are listed in Tables 3 and 4. The overall trend of brain and spinal tumors were increasing. Number of brain tumors almost doubled from 17 (6.5%) cases in 2009 to 29 (11.2%) cases in 2010. Following 2010 most frequent brain tumors were identified in 2013 and 2011 with the frequency of 24 (9.3%) and 22 (8.5%). This increasing trend was more dramatic for spinal tumors and the identified cases were almost quadrupled from 2 (3.5%) cases in 2009 to 8 (14%) cases in 2010. Following 2010 spinal tumors were most frequent in 2011 and 1998 with frequency of 7 (12.2%) and 6 (10.5%), respectively. Detailed trends of brain and spinal tumors are illustrated in Figures 1 and 2.

**Table 1:** Detailed Distribution Of Brain Tumors between Genders

**Tumor category**	**Female N (%)**	**Male N (%)**
Ependymoma	19 (59.4)	13 (40.6)
Ependymoblastoma	3 (60)	2 (40)
Choroid plexus papilloma	4 (50)	4 (50)
Astrocytomas	11 (37.9)	18 (62.1)
Pilocytic astrocytoma	18 (64.3)	10 (35.7)
Glioblastoma multiforme	5 (55.6)	4 (44.4)
Oligodendroglioma	0 (0)	1 (100)
Unspecified gliomas	7 (87.5)	1 (12.5)
Astroblastoma	0 (0)	1 (100)
Mixed neuronal-glial tumors	3(42.8)	4 (57.2)
Medulloblastoma	16 (30.2)	37 (69.8)
PNET	8 (50)	8 (50)
Atypical teratoid/rhabdoidtumor	1 (33.3)	2 (66.7)
Craniopharyngioma	9 (52.9)	8 (47.1)
Pineal parenchymal tumors	2 (40)	3 (60)
Meningioma	4 (44.4)	5 (55.6)
Angioblastic meningioma	1 (100)	0 (0)
Atypical meningioma	2 (100)	0 (0)
Lymphoma	1 (25)	3 (75)
Metastasis	7 (36.9)	12 (63.1)
Tumor category	Female n (%)	Male n (%)
Ependymoma	19 (59.4)	13 (40.6)
Ependymoblastoma	3 (60)	2 (40)
Choroid plexus papilloma	4 (50)	4 (50)
Astrocytomas	11 (37.9)	18 (62.1)
Pilocytic astrocytoma	18 (64.3)	10 (35.7)
Glioblastoma multiforme	5 (55.6)	4 (44.4)
Oligodendroglioma	0 (0)	1 (100)
Unspecified gliomas	7 (87.5)	1 (12.5)
Astroblastoma	0 (0)	1 (100)
Mixed neuronal-glial tumors	3(42.8)	4 (57.2)
Medulloblastoma	16 (30.2)	37 (69.8)
PNET	8 (50)	8 (50)
Atypical teratoid/rhabdoidtumor	1 (33.3)	2 (66.7)
Craniopharyngioma	9 (52.9)	8 (47.1)
Pineal parenchymal tumors	2 (40)	3 (60)
Meningioma	4 (44.4)	5 (55.6)
Angioblastic meningioma	1 (100)	0 (0)
Atypical meningioma	2 (100)	0 (0)
Lymphoma	1 (25)	3 (75)
Metastasis	7 (36.9)	12 (63.1)

**Table 2:** Age of Diagnosis -Brain Tumors

**Tumor category**	**Age Mean±(SD) (yr)**
Ependymoma	4.3±(4)
Ependymoblastoma	2±(2.2)
Choroid plexus papilloma	0.6±(0.3)
Astrocytomas	6.2 ±(3.4)
Pilocytic astrocytoma	5.6±(3.5)
Glioblastoma multiforme	6.6±(4.8)
Oligodendroglioma	8.00±(0)
Unspecified gliomas	4.4±(3.7)
Astroblastoma	1.00 0 ±(0)
Mixed neuronal-glial tumors	7.6 ±(4.7)
Medulloblastoma	5.4 ±(3.5)
PNET	4.2 ±(3.5)
Atypical teratoid/rhabdoidtumor	1.5 ±(0.8)
Craniopharyngioma	9.0±(2.6)
Pineal parenchymal tumors	12 ±(2.1)
Meningioma	12±(2.2)
Angioblastic meningioma	6 ±(0)
Atypical meningioma	0.2 ±(0.1)
Lymphoma	3.8 ±(2.8)
Metastasis	4.6±(1.3 )

**Table 3:** Detailed Distribution of Spinal Tumors Between Genders

**Tumor category**	**Female n (%)**	**Male n (%)**
Ependymoma	0 (0)	1 (100)
Ependymoblastoma	0 (0)	1 (100)
Astrocytomas	3 (60.0)	2 (40.7)
Pilocytic astrocytoma	1 (50)	1 (50)
Unspecified gliomas	3 (100)	0 (0)
PNET	2 (28)	5 (72)
Meningioma	2 (33.3)	4 (66.7)
Neuroblastoma	3(42.9)	4(57.1)
Ganglioneuroblastoma	0 (0)	2 (100)
Ganglioneuroma	2 (100)	0 (0)
Spindle cell tumor	0 (0)	1 (100)
Hemangioblastoma	1 (100)	0 (0)
Metastasis	11(57.9)	8(42.1)

**Table 4:** Age of Diagnosis -Spinal Tumors

**Tumor category**	**Age Mean±(SD) (yr)**
Ependymoma	0.08±(0)
Ependymoblastoma	4±(0)
Astrocytomas	6.5±(5.5)
Pilocytic astrocytoma	7±(6.1)
Unspecified gliomas	6.6±(3.7)
PNET	5.3±(3.4)
Meningioma	12.9±(1.5)
Neuroblastoma	1.9± (0.7)
Ganglioneuroblastoma	4±(0)
Ganglioneuroma	5±(0.7)
Spindle cell tumor	6±(0)
Hemangioblastoma	11±(0)
Metastasis	3.2±(2.3)

## Discussion

In this study, we retrospectively reviewed 315 cases of confirmed CNS tumors admitted to Mofid Pediatric Hospital in the last 18 years. Our findings regarding the most common brain and spinal tumors, gender distribution and age of diagnosis were comparable with other national and global reports. According to statistics published by SEER Program of the National Cancer Institute in the United States, CNS tumors are the leading solid malignancy during childhood (11). Male preponderance is a known fact in pediatric brain tumors (12). Sexual dimorphism in tumor biology could account for this gender disparity and detailed mechanisms regarding role of sex hormones and sex chromosomes on brain tumorigenesis are areas of ongoing investigations (13). Previous studies in Iran reported 1.2 and 1.68 male to female ratio in pediatric brain tumors (14, 15). In our study, male to female ratio was 1.2 for brain tumors, which is comparable with Australian (16), German (17), Canadian (18) and US studies, but is less than 1.6 ratio reported in a large single center study in China (6). In this study, malignant CNS tumors were most common in 1-4 yr age group. In annual age-adjusted rates published based on SEER database, most common age of diagnosis of pediatric brain tumors were 1-4 yr age group as well (19). The four most common brain tumors in our study were astrocytomas, medulloblastoma, ependymoma and craniopharyngioma. This pattern matches the largest national report (14), a recent single center study in Australia (16) and is comparable with the retrospective report of 1485 cases in China (6), with higher frequency of medulloblastoma in our subjects. High frequency of low-grade astrocytic tumors in our subjects also matches the published studies on pediatric brain tumors (20, 21). However, changes in pathological classification of pediatric CNS tumors have played a remarkable role in observed variations and apparent changes of trend of some tumor categories, particularly pilocytic astrocytoma (22). The observed frequency of ependymoma was also compatible with established reports that estimate an approximate 10% prevalence of ependymomas in pediatric intracranial tumors. In subjects under 3 years of age, the vast majority of ependymomas were also located at the posterior fossa, which is in line with the typical demographics of this tumor category in this age group (23). Medulloblastoma comprised 20.2% of brain tumors in our sample and this finding is also in line with epidemiologic investigations that highlight similar frequency of this tumor category as the most common malignant pediatric CNS tumor (7). Much lower frequency of medulloblastoma was reported by a large single center study in Australia, which is noteworthy (16). Moreover, following astrocytic tumors and medulloblastoma, craniopharyngioma was the most common tumor in our sample and this pattern of frequency matches most studies in pediatric brain malignancies (24). Review of the literature suggests that about onehalf to two-thirds of pediatric brain tumors are supratentorial. Statistics based on Central Brain Tumor Registry of the US (CBTRUS) data, highlight an even distribution of supratentorial and infratentorial tumors in children (20). In our subjects, 53.1% of the tumors were supratentorial, which is lower than the 62% rate observed in the Chinese single center study (6). In contrast to these findings, majority of tumors (67.7%), were infratentorial in a recent study on cases admitted to MAHAK’s Pediatric Cancer Treatment and Research Center in Iran (15). Gliomas, PNET and neuroblastma were the most primary spinal tumors in our study. This is comparable with another study in a large referral center study that reported neurodevelopmental tumors, astrocytomas and neuroblastomas as the most common diagnostic categories in a retrospective review (25). However, the unusual high frequency of neurdevelopmental tumors including dermoid tumors and teratomas in that study is in contrast to several published reports. The most common spinal tumors in the Chinese study were ependymal tumors and schwanomas followed by astrocytic tumors (6). Similar to Wilson et al. (25) we found very low frequency of ependymal tumors compared to similar single center studies (6, 26). Our subjects were younger than 15 years old and it is well established that ependymomas of the spinal cord occur more often in the adult population (27). This observed disparity between various reports on demographics of pediatric spinal tumors has been attributed to the low prevalence of spinal masses in children (28). Moreover, we did not observe a gender predilection in spinal tumors, which is in line with reports on this topic in the literature (28, 29). Spinal metastases were mostly caused by neuroblastoma, germ cell tumors, Ewing’s sarcoma and rhabdomyosarcoma that could be compared with previous studies (30). The increasing trend observed in this retrospective review should be interpreted with caution and in the context of possible referral biases introduced by the single center nature of this study. However, it could warrant further analyses at provincial and national levels to detect probable changes in the overall trend of pediatric CNS tumors. The existing literature highlights the improvements in the diagnostic sensitivity due to increased availability of magnetic resonance imaging as a crucial contributor in this apparent increasing trend of CNS tumors (22). In addition, factors like changes of tumor classifications and increased exposure to the known and unidentified environmental risk factors should be considered and thoroughly investigated as other possible contributing factors. 

**In conclusion**, in this retrospective 18-year review in a large referral pediatric center, we described demographics of CNS tumors in 315 subjects. Our results on demographics are comparable with similar single center studies on this topic with disparities that could be attributed to the single center nature of our study and geographical, environmental and racial variations in childhood CNS tumors. The increasing trend of pediatric CNS tumors could warrant further investigations at provincial and national levels to investigate probable contributing environmental risk factors. Furthermore, the demographic data presented in this study could be used as a reliable profile to set priorities for rationing the limited resources, particularly at large referral pediatric hospitals.

## Authors’ Contribution

HAG collected the data, designed the study, analyzed the data and drafted the manuscript. HH helped with methodological issues and revised the manuscript. AF and HM operated the patients, collected pathologic samples and revised the manuscript. YN and MK helped with pathologic assessment of samples supervised diagnostic accuracy and data gathering. FJ supervised the study design, checked reliability of collected data, helped in drafting and revision of the manuscript.
